# Examining the effects of national initiatives to improve the physical health of people with psychosis in England: secondary analysis of data from the National Clinical Audit of Psychosis

**DOI:** 10.1192/bjb.2021.38

**Published:** 2022-06

**Authors:** Ryan Williams, Sagana Natkulasingam, Beatrice Tooke, Ella Webster, Alan Quirk, Veenu Gupta, Paul French, Jo Smith, Mike J. Crawford

**Affiliations:** 1 Royal College of Psychiatrists, UK; 2 Imperial College London, UK; 3 University of Liverpool, UK; 4 University of Worcester, UK

**Keywords:** Physical health, psychosis, schizophrenia, community mental health teams, early intervention

## Abstract

**Aims and Methods:**

To examine whether national initiatives have led to improvements in the physical health of people with psychosis. Secondary analysis of a national audit of services for people with psychosis. Proportions of patients in ‘good health’ according to seven measures, and one composite measure derived from national standards, were compared between multiple rounds of data collection.

**Results:**

The proportion of patients in overall ‘good health’ under the care of ‘Early Intervention in Psychosis’ teams increased from 2014–2019, particularly for measures of smoking, alcohol and substance use. There was no overall change in the proportion of patients in overall ‘good health’ under the care of ‘Community Mental Health Teams’ from 2011–2017. However, there were improvements in alcohol use, blood glucose and lipid levels.

**Clinical implications:**

There have been modest improvements in the health of people with psychosis over the last nine years. Continuing efforts are required to translate these improvements into reductions in premature mortality.

Concerns have repeatedly been raised regarding premature mortality among people with schizophrenia and other psychotic disorders.^1–6^ People who experience psychosis die on average 10–20 years earlier than the general population.^7,8^

Factors contributing to this inequality may include economic disadvantage, health risk behaviours and difficulties accessing and adhering to medical treatments.^9–11^ These frequently translate to poor physical health, and psychotic disorders are strongly associated with unfavourable outcomes for a range of physical health measures: smoking status, weight, serum glucose levels, blood pressure and serum lipids.^12,13^ These issues can be compounded by metabolic side-effects of antipsychotic medication,^14–17^ and an increased risk of cardiovascular disease is widely accepted as the primary mediator of reduced life expectancy.^4,18–20^

## National initiatives

In 2014, the National Institute for Health and Care Excellence (NICE) produced recommendations to guide screening and intervention for common physical health problems experienced by people with psychosis.^21^ The same year, NHS England announced a new Commissioning for Quality and Innovation (CQUIN) framework, providing financial incentives for physical health screening and interventions within secondary mental health services.^22^ The ‘Positive Cardiometabolic Health Resource’ was published, with support from the Royal Colleges of Psychiatrists, Physicians, Nursing and General Practice, establishing a user-friendly manual for clinicians providing care to patients with severe mental illness.^23^

Since these initiatives were implemented, there is some evidence that the quality of physical healthcare delivered to patients with psychosis has improved.^24^ However, there are ongoing concerns that this has not translated to an improvement in patients’ health. A recent study found that cardiometabolic risk factors were already pronounced in those presenting to services with a first episode of psychotic illness, and that physical health deteriorated during the first year of treatment.^25^

In light of these apparent contradictions, we aimed to investigate whether physical health has improved among people with psychotic disorders. We conducted a secondary analysis of data gathered over the past 9 years, during national audits of services for people with psychosis.

## Method

All data for this study were collected during two audit rounds conducted as part of the National Clinical Audit of Psychosis (NCAP) by the Royal College of Psychiatrists.^26^ Both examined services providing care to people with psychotic disorders in England. A ‘core’ audit generated data on people under the care of community mental health teams (CMHTs), with three rounds of data collection in 2011, 2013 and 2017. A ‘spotlight’ audit collected additional data on the quality of care received by people with first-episode psychosis (FEP) who received care from early intervention for psychosis (EIP) teams. Three rounds of the spotlight audit were conducted in 2014, 2018 and 2019. All National Health Service (NHS) Provider Trusts in England with CMHTs and EIP teams that provided care to patients with psychotic disorders were invited to take part in these respective audits.

For both audits, all participating organisations were asked to provide an anonymised list of eligible patients who fulfilled inclusion criteria during a 12-month ‘sampling window’ before the point of data collection. From each list, 100 patients were randomly selected for inclusion in the audit. For the CMHT audit, patients were eligible for inclusion if they had an active period of care with a participating CMHT, were aged ≥18 years and had an established diagnosis of schizophrenia or schizoaffective disorder (ICD-10 codes F20/F25) recorded during the 12-month sampling period. Patients were excluded if they had received in-patient care or care from an EIP team during this period. For the EIP audit, patients were eligible for inclusion if they had an active period of care with a participating EIP team, were aged ≥14 years and had a diagnosed ‘first episode’ of any psychotic disorder (ICD-10 codes F20-F29) recorded during the 12-month sampling period. For the purposes of this study, we excluded any patients whose host organisation did not participate in all three rounds of the respective audit.

For both audits, staff from each organisation were asked to conduct a retrospective review of case notes from the sampling window, and extract data to complete an online data collection tool. The tool included questions on physical health measures, quantifying patients’ smoking status, alcohol use, blood pressure, body mass index (BMI), serum glucose, serum lipids and whether they were known to use illicit substances. The data collection tools for both audits were based on NICE guidance for management of psychotic disorders.^21^ They were developed in collaboration with patients and providers of psychiatric services, and carer representatives with lived experience of supporting patients with psychotic disorders. The tool was piloted by six volunteer trusts before the main audit, to ensure that the process was understandable and acceptable.

During the development of this project, the National Research Ethics Service and the Ethics and Confidentiality Committee of the National Information Governance Board advised that formal ethical approval and individual participants' informed consent were not required because this was a secondary analysis of audit data and patient-identifiable data were not being collected. The authors assert that all procedures contributing to this work comply with the ethical standards of the relevant national and institutional committees on human experimentation and with the Helsinki Declaration of 1975, as revised in 2008.

### Exposure, outcome measures and covariates

The primary outcome measures for this study were whether patients were considered to be in ‘good health’, according to for seven discrete physical health measures and one composite measure. The seven measures of good health were smoking status (not currently smoking, e.g. non-smoker or ex-smoker), alcohol use (no recorded ‘harmful or hazardous’ alcohol use), illicit substance use (no recorded illicit substance use), blood pressure (normotensive, i.e. <140/90 mmHg), BMI (within normal range, i.e. 18.5–24.9), serum glucose (within normal range, i.e. fasting blood glucose <5.5 mmol/L and/or random plasma glucose <11.1 mmol/L and/or hemoglobin A1C <42 mmol/mol) and serum lipids (within normal range, i.e. total serum cholesterol <5.1 mmol/L and/or high-density lipoprotein >1 mmol/L and/or non-high-density lipoprotein <4.1 mmol/L). The definition of good health for each measure was based on the standards implemented by the national Mental Health Commissioning for Quality and Innovation analysis.^22^ To be considered in good health for the composite measure, patients had to fulfil the criteria for good health for all of the seven discrete measures.

In addition, patients’ age and gender were recorded, to provide demographic information about the overall sample for each audit.

### Statistical methods

We used SPSS (version 26 for Windows)^27^ to analyse the study data. For each round of the audits, the proportion of patients with good health were calculated for each of the physical health measures and the composite measure. The variation in these proportions between each round of the two audits was then examined with binomial logistic regression.

Variation in demographic characteristics (age and gender) were compared between the CMHT and EIP audits, using *t*- and *χ*^2^-tests, respectively.

For many patients, data were not recorded for some of the physical health measures (presumably because it was not available in the clinical records, possibly because of patients refusing to undergo investigation or provide information).^28^ Missing values were not included in the analysis.

## Results

For the CMHT audit, 57 NHS Provider Trusts submitted data for all three rounds. Data from 16 752 sets of case notes were analysed (4618 from the first round in 2011, 4785 from the second round in 2013 and 7349 from the third round in 2017). For the EIP audit, 54 NHS Provider Trusts submitted data for all three rounds. Data from 20 611 sets of case notes were analysed (2158 from the first round in 2014, 8768 from the second round in 2018 and 9685 from the third round in 2019).

Table 1 summarises the demographic characteristics (age and gender) for the total samples of the CMHT audit and EIP audit, respectively. Across the three rounds, patients in the CMHT audit were significantly older than those in the EIP audit, with mean ages of 47.11 years and 29.66 years, respectively (*t*(37 361) = 156.94, *P* < 0.001). In the CMHT audit, 65.6% of the total sample were men, compared with 64.2% in the EIP audit, which was not a statistically significant difference.

**Table 1:** Demographic characteristics of people with psychosis in the CMHT and EIP audits

	CMHT audit	EIP audit
Age, mean (s.d.)	47.11 (±12.02)	29.66 (±9.47)
Difference in age between audits was statistically significant as determined by *t*-test: *t*(37361) = 156.94, *P* < 0.001		
Gender, *n* (%)		
Male	10 989 (65.6)	13 232 (64.2)
Female	5763 (34.4)	7379 (35.8)
Difference in gender between audits was not statistically significant as determined by *χ*^2^-test: *χ*^2^ = 1.23, *P* = 0.267		

CMHT, community mental health team; EIP, early intervention in psychosis.

Table 2 summarises the proportion of CMHT patients in good health according to each of our outcome measures (including the composite measure), and the variation in these proportions over time across the three rounds of the CMHT audit. There were variable amounts of missing data for each of the seven outcome measures, meaning that the composite measure could only be used for 31.3% (5243/16 752) of CMHT patients.

**Table 2:** Proportion of people with psychosis with good health outcomes at each round of the community mental health team audit

	2011 Audit	2013 Audit	2017 Audit
	*n/N*	*n/N*	*n/N*
	%	%	%
	Odds ratio (95% CI), *P*-value	Odds ratio (95% CI), *P*-value	Odds ratio (95% CI), *P*-value
Smoking	1694/4016	1769/4286	2784/6342
	42.2	41.3	43.9
	Reference	0.97 (0.89–1.06), 0.566	1.07 (0.99–1.16), 0.090
Alcohol use	2691/3197	2887/3387	5686/6410
	84.2	85.2	88.7
	Reference	1.09 (0.95–1.24), 0.244	1.48 (1.31–1.67), <0.001
Illicit substance use	3377/3888	3699/4243	5281/6332
	86.9	87.2	83.4
	Reference	1.03 (0.90–1.17), 0.689	0.76 (0.68–0.85), <0.001
Body mass index	502/1202	571/2587	992/4537
	22.8	22.1	21.9
	Reference	0.96 (0.84–1.10), 0.572	0.95 (0.84–1.07), 0.406
Blood pressure	1900/2593	2191/2946	3642/4855
	73.3	74.4	75.0
	Reference	1.06 (0.94–1.19), 0.371	1.10 (0.98–1.22), 0.107
Blood glucose levels	1449/2297	1502/2690	3393/4332
	63.1	55.8	78.3
	Reference	0.74 (0.66–0.83), <0.001	2.12 (1.89–2.36), <0.001
Blood lipids	924/2186	1143/3002	2350/4152
	42.3	42.3	56.6
	Reference	1.00 (0.89–1.12), 0.998	1.78 (1.60–1.98), <0.001
Composite measure	33/1004	34/1372	76/2867
	3.3	2.5	2.7
	Reference	0.74 (0.46–1.22), 0.294	0.80 (0.53–1.21), 0.348

There was some evidence of improvement in health. CMHT patients in the third round were significantly more likely than those in the first round to be in good health according to measures of alcohol use (odds ratio 1.48, 95% CI 1.31–1.67, *P* ≤ 0.001), blood glucose levels (odds ratio 2.12, 95% CI 1.89–2.36, *P* < 0.001) and blood lipids (odds ratio 1.78, 95% CI 1.60–1.98, *P* < 0.001).

However, CMHT patients in the third round were less likely to be in good health for the illicit substance use measure (odds ratio 0.76, 95% CI 0.68–0.85, *P* < 0.001), i.e. a higher proportion of CMHT patients were using illicit substances in 2017 compared with 2011. The proportion of CMHT patients in overall good health according to the composite measure was consistently low across all three rounds of the audit, and decreased from 3.3% in 2011 to 2.7% in 2017, although this was not statistically significant.

Table 3 summarises the proportion of EIP patients in good health according to each of our outcome measures (and the composite measure), and the variation in these proportions over time across the three rounds of the EIP audit. Similarly, there were variable amounts of missing data for each of the seven outcome measures, meaning that the composite measure could only be used for 56.4% (11 625/20 611) of EIP patients.

**Table 3:** Proportion of people with psychosis with good health outcomes at each round of the audit of early intervention in psychosis services

	2014 Audit	2018 Audit	2019 Audit
	*n/N*	*n/N*	*n/N*
	%	%	%
	Odds ratio (95% CI), *P*-value	Odds ratio (95% CI), *P*-value	Odds ratio (95% CI), *P*-value
Smoking	885/1808	4015/7832	4581/8487
	49.0	51.3	54.0
	Reference	1.10 (0.99–1.22), 0.080	1.22 (1.11–1.35), <0.001
Alcohol use	1620/1853	7021/7774	7749/8526
	87.4	90.3	90.9
	Reference	1.34 (1.15–1.57), <0.001	1.43 (1.23–1.68), <0.001
Illicit substance use	1182/1905	5711/7831	6410/8517
	62.1	72.9	75.3
	Reference	1.65 (1.48–1.83), <0.001	1.86 (1.68–2.07), <0.001
Body mass index	460/1044	2314/6667	2662/7566
	44.1	34.7	35.2
	Reference	0.68 (0.59–0.77), <0.001	0.69 (0.60–0.79), <0.001
Blood pressure	924/1106	5513/6733	6314/7750
	83.5	81.9	81.5
	Reference	0.89 (0.75–1.06), 0.195	0.87 (0.73–1.03), 0.104
Blood glucose levels	721/803	5071/5525	6175/6733
	89.8	91.8	91.7
	Reference	1.27 (0.99–1.63), 0.068	1.26 (0.99–1.61), 0.075
Blood lipids	506/741	3546/5416	4265/6564
	68.3	65.5	65.0
	Reference	0.88 (0.75–1.04), 0.141	0.86 (0.73–1.01), 0.079
Composite measure	37/1543	422/4465	576/5617
	2.4	9.5	10.3
	Reference	4.25 (3.02–5.98), <0.001	4.65 (3.32–6.52), <0.001

This audit also showed improvements in some of the measures of health over time: notably, those relating to smoking (odds ratio 1.22, 95% CI 1.11–1.35, *P* < 0.001), alcohol use (odds ratio 1.43, 95% CI 1.23–1.68, *P* < 0.001) and illicit substance use (odds ratio 1.86, 95% CI 1.68–2.07, *P* < 0.001). Furthermore, the proportion of EIP patients with overall good health was significantly higher in the third round compared with the first (odds ratio 4.65, 95% CI 3.32–6.52, *P* < 0.001), although this remained the minority (10.3%). Also, EIP patients in 2019 were significantly less likely than those in 2014 to be in good health for the BMI measure (odds ratio 0.69, 95% CI 0.60–0.79, *P* < 0.001).

## Discussion

This study corroborates previous findings that the physical health of people with psychosis remains poor, despite an improvement in physical health screening and intervention following national initiatives implemented in England since 2014.^24^

Both CMHT and EIP audits showed improvements in health according to some of these measures, and deteriorations in others. The proportion of patients in overall good health according to a composite measure was low across all rounds of both audits, but did improve significantly post-2014 for those patients receiving care from EIP services.

Unhealthy weight remains a particular area of concern, with large proportions of patients in poor health according to BMI across all rounds of both audits, and a significant deterioration over time in the EIP audit. This supports existing evidence that weight management is challenging for patients with psychotic disorders.^29,30^

We did find some evidence of a modest improvement in rates of smoking amongst patients under the care of EIP services post-2014. This contradicts recent studies where results have been more pessimistic,^25,31^ but would be in line with previous research suggesting a change in the epidemiology of smoking, with a gradual reduction in smoking in younger age groups.^32^ This improvement over time was not reflected in the CMHT audit, where patients were on average significantly older.

There was also a marked reduction post-2014 in the proportion of patients under the care of EIP services who were using illicit substances. This contrasted with the CMHT audit where the proportion increased slightly over time.

### Strengths and limitations

Data were obtained from large, heterogenous samples over a 9-year period: all NHS Trusts in England with CMHT and EIP services that provided care to patients with psychotic disorders were invited to participate in the respective audits. These data therefore represent a variety of different settings, and we would expect that results would be generalisable to similar patient groups across the country. The primary outcome measures we used to assess physical health are universally recognised as clinically important, and the thresholds for good health were based on national standards that have been widely used elsewhere.^33–35^

However, this study does have important limitations. First, this is an observational study and we do not know what caused the changes we observed. Although they may reflect changes in services during this period, other changes in society are affecting population health,^36^ and these could be responsible for some or all the differences we detected.

The EIP and CMHT audits were conducted at different times, both before and after the CQUIN framework was implemented, and used different selection criteria. Differences in the physical health of patients between the two are therefore likely to reflect the differing clinical and socioeconomic demographic characteristics of the patients in each audit, as well as different processes of care. We were able to examine changes in physical health over time within each audit, by comparing different rounds. However, as each service provided a random sample of eligible patients at each round, subsequent rounds of the same audit did not necessarily include the same cohort, and so we are unable to make inferences about changes in health at the level of individual patients even within the same audit.

For both the EIP and CMHT audits, we were able to examine physical health measures before and after the introduction of the Commissioning for Quality and Innovation programme.^22^ Although the introduction of the programme was associated with a marked increase in the proportion of patients who were offered interventions for their physical health,^24^ we found only limited evidence that this resulted in changes to the health of people under the care of CMHTs.

These data were produced from retrospective case note audits at each round, and are therefore dependant on accurate reporting and documentation of events at the time of occurrence. Clinicians working in CMHTs and EIP services may not have had full access to records held by primary care, where some physical health screening may have occurred.

Also, there were variable amounts of missing data for each primary outcome measure, meaning that the composite measure could only be used for a relatively small proportion of the overall sample. However, the proportion of missing data did generally improve over subsequent rounds of each audit, possibly reflecting the previously noted incentivised improvements in screening rates and recording.^24^

### Implications

We found some evidence that overall health improved for patients with psychotic disorders under the care of EIP services between 2014 and 2019. However, we did not find evidence of a similar improvement for patients under the care of CMHTs between 2011 and 2017.

This may reflect differences in the model of care implemented by EIP services. Typically, these services advocate a more ‘intensive’ programme of intervention, with a focus on relatively smaller case-loads, proactive engagement and an increased frequency of contact.^35–39^ Although this approach is primarily intended to address psychiatric symptoms, multiple sources have suggested additional benefits for patients’ overall health, including increased levels of screening for physical health problems.^40^ EIP services may, therefore, have been better placed to implement changes resulting from the national initiatives rolled out in 2014.

However, the difference may also be because of differences in demographic and clinical characteristics between patients in the CMHT audit and the EIP audit. Patients in the EIP audit were younger on average, and were also likely to have had a shorter duration of psychiatric symptoms (as this audit specifically examined patients with a diagnosed ‘first episode’ of a psychotic disorder). These patients may have been more accepting of interventions intended to improve their physical health and, therefore, have seen greater improvements – even if CMHTs and EIP services had implemented identical changes since 2014.

It may even be the case that people in younger age groups have become more ‘health conscious’ in recent years regardless of any intervention, as suggested by some epidemiological studies.^32^ However, this seems unlikely in these cohorts in light of previous findings that patients on EIP case-loads already had pronounced cardiovascular risk factors, even at the point of first presentation.^25^

Both audits identified some isolated areas of improvement in specific measures. Interestingly, these differed between the patients in the CMHT audit, where there were improvements related to alcohol use, serum glucose level and lipids, and the EIP audit, where there were improvements in alcohol use, smoking and illicit substance use. Again, these changes may relate to demographic differences in the patients under the care of these respective services. Older patients may be more receptive to those that they perceive as reducing their risk of major cardiovascular events, such as reduction in serum glucose and lipids.

However, these varying improvements may also be because of differences in the treatment approaches adopted by CMHTs and EIP services. These findings merit further research to identify the factors explaining these variations, as well as the improvement in overall good health seen among those treated by EIP services. There may be elements where each service outperforms the other; for example, access to staff with training in physical health interventions, or improved links with primary care. Cohort or case–control studies could be used within CMHTs and EIP services to examine what interventions are associated with favourable physical health outcomes at an individual patient level. Qualitative studies to explore the perspectives of patients with psychosis may also identify effective strategies for improving patients’ physical health. It may also be of interest to include other services, such as assertive outreach teams, in future studies.

The fact that the EIP audit showed an improvement in rates of illicit substance use over time, but the CMHT audit showed the opposite, is an intriguing finding and of unclear significance. It does not seem to reflect the current understanding of the changing epidemiology of illicit substance use,^41^ and suggests that EIP services have been able to implement effective measures to support people in abstaining from illicit substances.

Interestingly, the one measure where both CMHTs and EIP services improved over time was alcohol use. Many drug and alcohol services in the UK are now provided by third-sector organisations rather than NHS mental health teams.^42,43^ The fact that both CMHTs and EIP services were able to effect improvement suggests that effective liaison with external organisations may be a key strategy for improvement, rather than attempting to deliver more services with internal infrastructure, which may already be overstretched.

Both audits showed specific areas where standards of physical health worsened over time. The findings related to patients’ weight are particularly concerning: the proportion of patients with a healthy BMI fell significantly across the three rounds of the EIP audit, and was universally low in the CMHT audit. Weight gain is a well-recognised and particular troubling side-effect of many antipsychotic medications.^17^ Given the implications of obesity for subjective well-being, medication adherence and therapeutic outcomes in the context of treatment for psychosis, and associated diabetes and cardiovascular risk and likelihood of premature mortality,^44–46^ improving interventions in this area needs to remain a focus for researchers. To date, studies of current weight management programmes for people with psychosis have produced mixed results.^47–49^

In addition to these findings, it is also important to note that the majority of patients did not have adequate information recorded for all seven of the physical health measures recommended by nationally agreed standards. Only around half of patients in the EIP audit, and a third of those in the CMHT audit, had usable data recorded for all seven measures. The reasons for this are unclear from this project, and may reflect barriers to physical health screening, such as refusal, lack of provision or inadequate documentation. Clearly, accurate monitoring is required, and will be the focus of quality improvement activities before future rounds of the national audits.

In conclusion, we found limited evidence that overall health improved for patients with psychotic disorders under the care of CMHTs, following the enactment of national initiatives in 2014, although there was some evidence of improvement in specific areas. There was more substantial evidence of improvements for those patients under the care of EIP services. This may be a result of differences in CMHT and EIP services’ abilities to implement effective changes in policy and procedures, or demographic and clinical differences in their respective patients. However, these findings merit further research into the processes underlying the improvements in health, to improve the standard of care for people with psychosis.

## Data Availability

All authors had access to the full study data-set. The data-set is held by the NCAP team at the College Centre for Quality Improvement, Royal College of Psychiatrists, and could be made available on request.

## About the authors

**Ryan Williams** is an NIHR Academic Clinical Fellow at the Department of Brain Sciences, Imperial College London, UK, and a Clinical Fellow with the College Centre for Quality Improvement, Royal College of Psychiatrists, UK. **Sagana Natkulasingam** is a medical student at the Faculty of Medicine, Imperial College London, UK. **Beatrice Tooke** is an NCAP Programme Manager with the College Centre for Quality Improvement, Royal College of Psychiatrists, UK. **Ella Webster** is an NCAP Programme Manager with the College Centre for Quality Improvement, Royal College of Psychiatrists, UK. **Alan Quirk** is Head of Clinical Audit and Research for the College Centre for Quality Improvement, Royal College of Psychiatrists, UK. **Veenu Gupta** is a Service User Advisor with the College Centre for Quality Improvement, Royal College of Psychiatrists, UK. **Paul French** is an Honorary Professor with the Department of Psychological Sciences, University of Liverpool, UK, and an NCAP EIP Clinical Advisor with the College Centre for Quality Improvement, Royal College of Psychiatrists, UK. **Jo Smith** is Professor of EIP and Psychosis at the School of Allied Health and Community, University of Worcester, UK, and an NCAP EIP Clinical Advisor with the College Centre for Quality Improvement, Royal College of Psychiatrists, UK. **Mike J. Crawford** is Professor of Psychiatry at the Department of Brain Sciences, Imperial College London, UK, and Director of the College Centre for Quality Improvement, Royal College of Psychiatrists, UK.
